# Clinical Outcome of Prefabricated Zirconia Crowns Cemented with Self-Adhesive Resin and Pure Glass Ionomer on Primary Teeth: A Retrospective Cohort Study

**DOI:** 10.3390/children11080991

**Published:** 2024-08-15

**Authors:** Murad Alrashdi, Atyaf Alhunti

**Affiliations:** 1 Department of Orthodontic and Pediatric Dentistry, College of Dentistry, Qassim University, Burayadh 52571, Saudi Arabia; 2 Pediatric Dentistry Department, Qassim Regional Dental Center, Qassim Health Cluster, Burayadh 52391, Saudi Arabia

**Keywords:** prefabricated zirconia crowns, self-adhesive resin cement, children

## Abstract

Retentive strength data are critical to predicting the long-term clinical performance of zirconia crowns for primary teeth. **Objectives**: This research assessed the clinical outcome of prefabricated zirconia crowns (PZCs) cemented with either self-adhesive resin cement or pure glass ionomer cement in the primary teeth of children. **Method**: In the present research, a sample of 162 prefabricated zirconia crowns were collected through convenience sampling. A follow-up examination was conducted at 12 and 24 months to assess the clinical outcomes of PZCs post cementation. **Results**: Zirconia crowns cemented with self-adhesive resin showed high clinical outcomes at both 12 and 24 months, with 95.1% of crowns retained. In contrast, crowns cemented with glass ionomer cement had slightly lower clinical outcomes, with 91.4% retained at 12 months and 84.0% retained at 24 months, indicating a significant difference (*p*-value). Long-term follow-up is crucial for the optimal maintenance of crown stability. **Conclusions**: Self-adhesive resin cement is a viable option for cementing PZCs in pediatric dentistry, demonstrating satisfactory clinical performance over both 12 and 24 months. Future studies comparing different types of cement are recommended for further validation of these results.

## 1. Introduction

Over the years, various restorative materials have been employed for primary teeth, each with its own advantages, disadvantages, indications, and contraindications [1]. Esthetics and function play important roles in the success of primary tooth restorations, as these are the primary concerns of parents [2]. Full-coverage coronal restorations achieved with prefabricated zirconia crowns (PZC) have become the preferred choice for primary teeth with extensive dental caries, owing to their superior esthetics, especially for the anterior teeth [3].

Fitting zirconia crowns for primary teeth requires only passive tooth reduction, which can result in lower clinical outcomes as compared to custom-made crowns. Furthermore, owing to the non-adhesive characteristics of the material, the cementation of zirconia crowns can be challenging [4]. Their retention status depends on the bond strength of the luting cement, which is influenced by the type of cement used [5]. Moreover, the cementation of PZCs is a technique- and moisture-sensitive procedure [6]. Glass ionomer and resin-based cements have superior bonding characteristics, which not only hold the crown in place but also reduce marginal microleakage [7].

Custom-made zirconia crowns are often cemented using conventional adhesive methods, with resin-based luting agents reportedly providing high clinical outcomes and the best marginal adaptation [8]. Although many studies have been performed to evaluate the retentive strength of zirconia crowns for permanent teeth, limited data are available for primary teeth. Various types of luting cement, such as glass ionomer cement (GIC), resin-modified GIC, resin cement, and bioceramic cement, have been used over the years for the cementation of PZCs [9]. However, there is limited literature supporting the use of any particular type of cement for primary teeth.

Resin cement was discovered in the early 1950s [10]. The acrylic resin, which is filled with silica particles, adheres to the enamel surface [11]. The advantages of resin cement include high bond and compressive strengths, low water solubility, and excellent esthetics [12]. Resin cement is categorized into two types—conventional resin cement with a separate adhesive system and self-adhesive resin cement. Self-adhesive resin cements consist of polymers that do not require etching or bonding to the tooth surface. Once mixed, the cement can be applied in a single step [13].

Retentive strength data are fundamental to predicting the long-term clinical performance of zirconia crowns for primary teeth [14]. To date, no universal method exists to improve the adhesion of cement to tooth surfaces. In a recent systematic review, Alrashdi et al. stated the need for further investigations on whether restoration failures are due to the type of cement used or the technique itself [15]. Previous clinical studies on zirconia crowns have typically involved short observation periods, relatively small samples, and have often been limited to a single type of self-adhesive cement. The purpose of this study was to assess the effectiveness of two distinct self-adhesive resin cements in the clinical outcomes of PZCs. The null hypothesis for this study is that there is no difference in clinical outcomes of PZCs cemented with different adhesive resin cements.

## 2. Materials and Methods

### 2.1. Ethics Approval

Ethics approval was obtained from the Institutional Review Board of Qassim University (protocol number: 23-20-09).

### 2.2. Study Design

A retrospective cohort study was conducted to assess the clinical outcome of PZCs cemented using self-adhesive resin cement and glass ionomer to the primary teeth of children. Dental records and charts were retrospectively searched for children who had received zirconia crowns. A comprehensive assessment of the crowns was carried out according to the modified California Dental Association criteria [16]. Each crown was evaluated for biological (recurrent caries, loss of vitality, and tooth fracture), technical (open margins, marginal overhang, under-contoured margins, crown fracture, porcelain chipping, the presence of crack lines, and an open contact), and esthetic complications (surface texture or smoothness, color mismatch, and marginal discoloration). However, the primary aim of this study was to assess the clinical outcomes related to the retention status of prefabricated zirconia crowns using two cements—self-adhesive resin and glass ionomer. The scope was narrowed to encompass only the retention status of crowns. The clinical performance of the zirconia crowns was assessed to analyze their clinical outcomes.

### 2.3. Study Sample

The study sample comprised mutilated, grossly carious, and/or large fillings requiring full-coverage coronal restoration with PZCs cemented using self-adhesive resin cement. In the present research, we used convenience sampling, screening the electronic records of all the patients visiting the clinics of the College of Dentistry at Qassim University from January 2021 to December 2023, and selecting those needing PZCs for at least one primary tooth. The sample size was calculated to ensure adequate power to detect differences in clinical outcomes, considering a significance level of 0.05 and a power of 0.80. All treated teeth in this study were anterior upper teeth, and no posterior teeth were included in the analysis. A total of 162 PZCs were included in the study. A follow-up appointment spanning a minimum of 12 months was employed to confirm the retention status of the crowns. Patients who did not attend this scheduled recall visit were excluded from the analysis. Re-cementation of crowns that became detached but could be reattached was documented. Instances in which crowns were not re-cemented typically involved conditions such as the presence of an infection necessitating tooth extraction, tooth mobility stemming from natural shedding, or trauma necessitating the extraction of the affected tooth.

### 2.4. Inclusion and Exclusion Criteria

The current study followed specific inclusion and exclusion criteria to include only the relevant and most appropriate cases, as follows: children aged 3 to 7 years, requiring full coronal restoration on primary teeth; children whose dental records and charts were available; and children receiving zirconia crowns cemented with either glass ionomer or self-adhesive resin. However, children who have not undergone full coronal restoration on primary teeth, children with incomplete records, those whose records did not specify the type of cement, and children with known allergies to dental materials were excluded.

### 2.5. Clinical Procedure

One pediatric dentist, trained to perform all the treatment steps described in the “NuSmile Zirconia crowns technical guide”, completed all the clinical procedures and outcome assessments. The teeth were prepared by performing passive tooth reduction, and the crowns were tried-in using NuSmile Try-In crowns (NuSmile, Houston, TX, USA). After drying the tooth and controlling bleeding, the cement was dispensed through the automix tip directly into the crown, which was positioned over the tooth and allowed to self-set for 20 s while maintaining gentle pressure on the crown. The buccal and lingual margins were tag-cured for 10 s each using a light-curing device (850–1000 mW/cm^2^), simplifying the removal of excess cement. The buccal and lingual surfaces were light-cured for an additional 10 s. Occlusion and bite were examined before and after final cementation. The cement used was RelyX™ Unicem, manufactured by 3M ESPE.

A similar procedure was followed for glass ionomer cement, Fuji II, which was manufactured by GC Corporation. The glass ionomer cement was mixed as per the instructions of the manufacturer and was directly applied into the crown. The buccal and lingual margins were tack-cured for 10 s each using a light-curing device (850–1000 mW/cm^2^, Elipar S10, 3M ESPE, St. Paul, MN, USA). For glass ionomer cement, the tooth surface was conditioned with polyacrylic acid for 10 s, rinsed, and dried before cement application.

### 2.6. Outcome Variable

In this study, the initial point of observation was defined as the moment of crown cementation, and the retention status time concluded with the occurrence of any clinical failure. Adhering to methodologies in prior studies, complications were categorized based on issues associated with the crown or the supporting tooth structure. The primary complication in this study was the debonding of the crown.

We defined cases as ‘successful’ in the absence of failures, if both the crown fit and the health of the tooth remained uncompromised. In contrast, ‘survival’ cases were those in which complications arose, but the continued use of the crown was feasible following straightforward remedial actions. An example of such a survival case would be a crown that debonded within the first week post cementation but was successfully recemented using the same adhesive material.

### 2.7. Explanatory Variables and Covariates

One explanatory variable was the type of cement used to fix the zirconia crown, either the self-adhesive resin cement or the GIC. Furthermore, the duration of follow-up after crown placement was used as an explanatory variable, as longer follow-up periods may reveal delayed or gradual changes in crown retention strength over time.

### 2.8. Statistical Analyses

Descriptive statistics was performed using IBM SPSS Statistics, v28 (IBM Corp, Armonk, NY, USA). Pearson’s chi-square tests were used to assess whether sex is associated with retention status. McNemar’s chi-squared test was used to evaluate the difference in the retention status of the two types of cement at the two different follow-ups. Kaplan–Meier survival analysis was used to determine crown retention status, with the log-rank (Mantel–Cox) test to assess factors impacting clinical outcome, including participant sex, age, and cement type. All statistical tests were two-tailed (α = 0.05).

## 3. Results

The demographic characteristics and clinical outcomes of the participants receiving zirconia crowns cemented with self-adhesive resin or glass ionomer cement (GIC) are summarized below. The majority of participants with zirconia crowns cemented with self-adhesive resin were aged 3 to 5 years, with 35.8% (*n* = 29) aged 3 years, 28.4% (n = 23) aged 5 years, and 19.8% aged 4 years, with an average age of 3.5, as shown in Table 1. Among participants with zirconia crowns cemented with self-adhesive resin, a slightly higher proportion were boys (55.6%) than girls (44.4%).

For zirconia crowns cemented with self-adhesive resin, the clinical outcomes at both 12 and 24 months were high, with 95.1% of crowns being retained at both time points. Conversely, crowns cemented with GIC showed slightly lower clinical outcomes, with 91.4% retention at 12 months and 84.0% retention at 24 months (Table 2). The debonding rate was higher for crowns cemented with GIC compared to those cemented with self-adhesive resin at both 12 and 24 months.

Pearson chi-square tests revealed that there was no statistically significant association between sex and retention status for either the self-adhesive resin or the glass ionomer groups at both 12 months and 24 months (Table 3).

**Figure 1:**
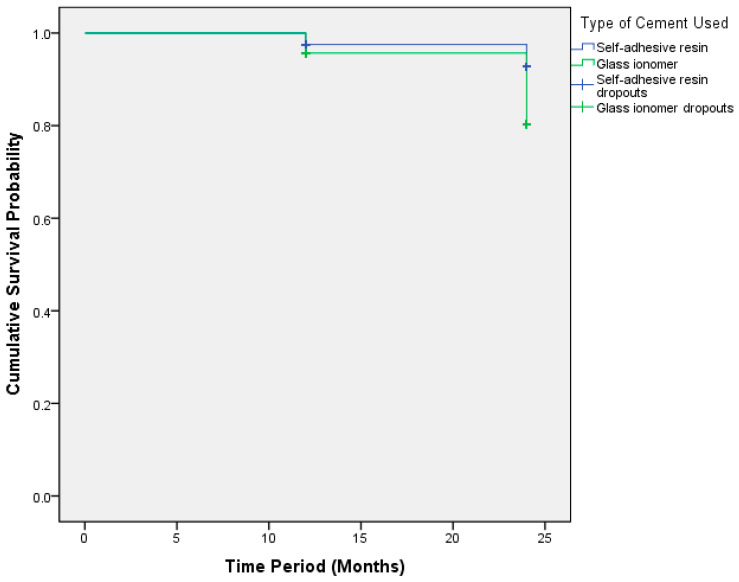
Kaplan–Meier clinical outcome for self-adhesive resin and glass ionomer at 12 and 24 month follow-up. Cum—cumulative. Censoring indicates that some individuals have not experienced the event (failure of dental cement) by the end of the study period or were lost to follow-up. The crosses indicate the censored data points.

A Mantel–Cox test was performed to compare the clinical outcome of the two different cements. The chi-square test statistic of 5.45 indicates a significant difference between the survival distributions of the different types of resin. The *p*-value of 0.019 suggests that the observed difference in survival distributions is statistically significant (Table 4). Therefore, this result provides strong evidence to conclude that the survival distributions for the different types of resin used are not equal, as illustrated in Figure 1. Some children with self-adhesive resin did not experience cement failure by the end of the study or were lost to follow-up, which is why they are marked as censored. Similarly, for some children using glass ionomer, the cement did not fail, which lead to censoring. Censoring reflects that not all subjects will complete the study or experience long-term follow-ups. In this way, the Kaplan–Meier method provides a more accurate estimate of survival probabilities.

A McNemar test indicated a *p*-value of 0.031, which shows the probability of observing the observed result (or a more extreme result) under the null hypothesis that the clinical outcomes at 12 and 24 months do not differ (Table 5).

## 4. Discussion

The aim of the present study was to retrospectively assess the clinical outcomes of zirconia crowns cemented with two different resins—self-adhesive resin and glass ionomer. Our results revealed that the majority of participants receiving zirconia crowns with self-adhesive resin were preschool-aged children, with a slightly higher proportion of boys than girls. This demographic distribution aligns with previous studies highlighting the prevalence of dental caries and the need for restorative interventions in young children.

Interestingly, we observed high clinical outcomes for crowns cemented with self-adhesive resin at both 12 and 24 months (95.1%), suggesting the effectiveness of this resin in maintaining crown stability over time. This rate aligns with the high success rates (up to 100%) observed in other studies using self-adhesive resin as cement [4,7].

Our study highlights the importance of appropriate surface treatment procedures and selecting the right cement to achieve a greater bond strength of zirconia crown in primary teeth. Prior to cementation, the tooth surfaces were carefully prepared to ensure the optimal adhesion of the crowns. For the glass ionomer cement (GIC) group, the tooth surface was conditioned using a 10% polyacrylic acid conditioner (GC Corporation, Tokyo, Japan) for 10 s. This step is critical as it cleans the tooth surface and enhances the bonding capability of the GIC by removing the smear layer and providing a suitable surface for chemical adhesion. The conditioned surface was then thoroughly rinsed with water and dried before the application of the GIC. In the case of the self-adhesive resin cement, RelyX™ Unicem (3M ESPE, St. Paul, Minnesota, USA), no separate conditioning agent was required as the cement itself contains functional monomers that promote adhesion to the tooth structure. The cement was directly applied to the prepared tooth surface, ensuring that the buccal and lingual margins were tack-cured for 10 s each using a light-curing device (850–1000 mW/cm^2^, Elipar S10, 3M ESPE).

In addition to comparing the outcomes of zirconium oxide crowns with those cemented using different materials, it is also important to consider studies that have examined other types of crowns in pediatric dentistry. For instance, several studies have evaluated the clinical performance of stainless steel crowns (SSCs), which are a well-established option in pediatric dentistry due to their durability and ease of placement. In comparison to zirconia crowns, SSCs have shown high retention rates but are often less preferred due to their esthetic limitations. Research by Walia et al. (2014) demonstrated that SSCs have a success rate ranging from 92% to 98% over a 24-month period, which is comparable to the retention rates observed in our study for zirconia crowns cemented with self-adhesive resin (95.1% at 24 months). However, the esthetic appeal of zirconia crowns makes them more desirable for anterior teeth, particularly in cases where parental satisfaction is a priority (Holsinger et al., 2016). Furthermore, studies involving pre-veneered crowns, which combine the durability of SSCs with an esthetic resin veneer, have reported success rates similar to zirconia crowns but with issues related to veneer chipping and wear over time (Randall, 2002). These findings suggest that while various crown materials can provide satisfactory clinical outcomes, the choice of material often depends on the balance between durability and esthetics, as well as patient and parent preferences. By comparing the outcomes of different crown materials, our study contributes to the broader understanding of the advantages and limitations of using zirconia crowns in pediatric dentistry, particularly when considering both esthetic and functional outcomes.

Despite these encouraging results, it is imperative to acknowledge the potential complications that could affect the zirconia-based restoration. Current laboratory and clinical studies have shown promising results, but the long-term outcomes are unclear. Our findings demonstrate the efficacy of self-adhesive resins for a durable crown retention status. However, limitations such as the study population and cement application techniques need to be considered [17]. Further research into these limitations is needed to gain a deeper insight into the long-term performance of self-adhesive resins.

In contrast, crowns cemented with glass ionomer exhibited slightly lower clinical outcomes (91.4% and 84.0% at 12 and 24 months, respectively), indicating potential limitations associated with this type of cement in ensuring a long-term crown retention status. While some studies have reported that the retention status of glass ionomer is comparable to that of resin-modified glass ionomer [18,19]. These variations from the current study could be associated with factors like patient-specific variables, cement type, and preparation techniques. However, the clinical outcome of glass ionomer cement was reported to be 82% to 100% [2,20,21,22]. Bioceramic cement achieved a 100% success rate in one study [6] but a much lower rate in another [23]. These discrepancies in the clinical outcome demonstrate the need to conduct a comprehensive evaluation to identify the most effective cement for PZCs, particularly considering the limitations of existing studies, which have limitations including inadequate details on sample sizes and confidence intervals.

The Pearson’s chi-square tests conducted in this study revealed no association between child gender and the retention status within either the self-adhesive resin or glass ionomer groups at 12 months and 24 months post treatment. These findings are consistent with a previous study that reported no statistically significant association between child gender and the clinical outcome of zirconia crowns [24]. However, another previous study suggested that it is important to consider clinical outcomes in light of potential confounding factors such as the surface area, height of the prepared crown, cementing medium, occlusal forces, and underlying dental conditions, which could have influenced crown clinical outcomes [25]. These factors were not considered in our study, so results regarding the clinical outcomes of zirconia crowns should be interpreted with caution.

Survival analysis using the log-rank (Mantel–Cox) test provided further insights into the differences in survival distributions between self-adhesive resin and glass ionomer. The significant difference suggested variations in the durability and longevity of crowns cemented with these two resins. It shows the importance of selecting the suitable cement material for the long-term retention of zirconia crowns in children. The McNemar test revealed a statistically significant difference in clinical outcomes at 12 and 24 months, underscoring the dynamic nature of crown retention status over time.

These results have important clinical implications for the selection of cement materials in pediatric restorative dentistry. Future studies may be conducted to investigate the underlying mechanisms contributing to the observed differences in clinical outcomes and exploring strategies to optimize long-term crown retention status in pediatric patients. Factors influencing PZC retention status, such as patient-specific factors (occlusion, oral hygiene, and dietary habits), technique sensitivity of cement application, and crown placement, should be discussed further [14,15]. Additionally, one major limitation of this study is the convenience sampling method that was used, which might have introduced a potential selection bias in the results. These factors must be considered when interpreting these results in clinical practice and future research.

Lastly, as pediatric dental materials and techniques continue to evolve, staying current with these developments is crucial. Innovative approaches, such as the use of newer biocompatible materials or minimally invasive techniques, may offer improved outcomes for pediatric patients. As such, ongoing research and clinical trials are essential in refining our understanding and application of these materials in pediatric dentistry.

## 5. Conclusions

This study demonstrates that zirconia crowns cemented with self-adhesive resin had a higher retention status at both 12 and 24 months compared to those cemented with glass ionomer cement. Statistical analyses confirmed significant differences in survival distributions between the two cements. The overall superior performance of self-adhesive resin suggests it may be a more reliable choice for pediatric zirconia crowns.

## Figures and Tables

**Table 1:** Age distribution of participants.

Variables	Mean Age (Years)	Range (Min–Max)	Std. Deviation
Age (years)	3.5	2–5	1.29

**Table 2:** Descriptive statistics of participants who had zirconia crowns.

Variables	Frequency (n)	Valid Percent	Std. Deviation
Age (years)			
2	13	16.0%	
3	29	35.8%	1.069
4	16	19.8%	
5	23	28.4%	
Sex			
Male	45	55.6%	0.500
Female	36	44.4%	
Retention status at 12 months (self-adhesive resin)			
Retained	77	95.1%	0.218
Debonded	4	4.9%	
Retention status at 24 months (self-adhesive resin)			
Retained	77	95.1%	0.218
Debonded	4	4.9%	
Retention status at 12 months (pure glass ionomer)			
Retained	74	91.4%	0.283
Debonded	7	8.6%	
Retention status at 24 months (pure glass ionomer)			
Retained	68	84.0%	0.369
Debonded	13	16.0%	

**Table 3:** Pearson chi-square tests for association between sex and retention status.

Cements	Value	df	*p* (Two-Sided)
Self-adhesive resin			
Retention status at 12 months	0.500	1	0.479
Retention status at 24 months	0.554	1	0.457
Glass ionomer			
Retention status at 12 months	0.053	1	0.819
Retention status at 24 months	0.655	1	0.422

**Table 4:** Log rank (Mantel–Cox) results.

Test	Chi-Square	df	*p*
Log rank (Mantel–Cox)	5.45	1	0.019

df—degrees of freedom.

**Table 5:** McNemar test results.

Parameter	N	*p* (Two-Tailed)
Retention status at 12 months and retention status at 24 months	162	0.031 ^1^

^1^ Binominal distribution used.

## Data Availability

Available based on request.
